# Dietary Inclusion of Monosodium Glutamate in Gestating and Lactating Sows Modifies the Preference Thresholds and Sensory-Motivated Intake for Umami and Sweet Solutions in Post-Weaned Pigs

**DOI:** 10.3390/ani9060336

**Published:** 2019-06-10

**Authors:** Sergio A. Guzmán-Pino, Cristian Lazcano, Valeria De Luca, Jaime Figueroa, Carolina Valenzuela, Eugeni Roura

**Affiliations:** 1 Departamento de Fomento de la Producción Animal, Facultad de Ciencias Veterinarias y Pecuarias, Universidad de Chile, Santa Rosa 11735, La Pintana, Santiago 8820000, Chile; valeria.deluca@ug.uchile.cl (V.D.L.); cvalenzuelav@u.uchile.cl (C.V.); 2 Agrícola Santa Lucía Ltda., Camino El Toco s/n, Pichidegua 2980000, Chile; clazcano@maxagro.cl; 3 Departamento de Ciencias Animales, Facultad de Agronomía e Ingeniería Forestal, Pontificia Universidad Católica de Chile, Vicuña Mackenna 4860, Macul, Santiago 7820436, Chile; figueroa.jaime@uc.cl; 4 Centre for Nutrition and Food Sciences, Queensland Alliance for Agriculture and Food Innovation, The University of Queensland, Hartley Teakle 83, St. Lucia, Brisbane, QLD 4072, Australia; e.roura@uq.edu.au

**Keywords:** consumption patterns, maternal diets, monosodium glutamate, post-weaned pigs, preference thresholds, sensory-motivated intake, sows’ milk, umami taste

## Abstract

**Simple Summary:**

Umami is an innately hedonic taste in pigs, which has been related to glutamic acid (i.e., monosodium glutamate (MSG)) and other dietary amino acids. We studied the inclusion of 5% MSG in gestating and lactating sow diets, with the objective of altering feeding behavior of the progeny. It was observed that the addition of MSG to maternal diets decreased the preference thresholds for umami and sweet tastants in the progeny, and increased sucrose sensory-motivated intake. In contrast, MSG supplementation decreased MSG-motivated intake and did not modify the total consumption and consumption patterns of pigs for these solutions. We concluded that MSG inclusion in maternal diets modifies feeding behavior in pigs in what appears to be a compensatory mechanism to balance the diet for basic nutrients.

**Abstract:**

Pigs show an innate preference for umami (monosodium glutamate, MSG) taste. Nevertheless, the influence of a pre and postnatal umami exposure remains unclear. An experiment was conducted to test the hypothesis that MSG inclusion into maternal diets would modify the feeding behavior of post-weaning pigs. A total of 22 sows were selected on day 85 of gestation and randomly assigned to one of two gestating and lactating programs (standard commercial diets without or with 50 g/kg of MSG). Later, 208 pigs born from these sows were selected to evaluate their preference thresholds, sensory-motivated intake, total consumption, and consumption patterns for MSG and sucrose solutions. Pigs born from MSG-fed sows showed lower (*p* < 0.045) preference thresholds for MSG and sucrose than did animals born from control sows, and displayed an increased (*p* < 0.050) sensory-motivated intake for sucrose and decreased for MSG. Conversely, no differences (*p* > 0.05) were observed in the total consumption or consumption patterns of MSG or sucrose solutions among pigs born from control and MSG-fed sows. It is concluded that the feeding behavior of nursery pigs can be influenced by pre and postnatal inclusion of a taste active compound into maternal diets. It would appear that a compensatory mechanism to balance dietary nutrients might be in place.

## 1. Introduction

In the wild, weaning in pigs is a long and progressive process that occurs between the 9th and 22nd week of age, allowing piglets a gradual transition from milk to solid foods [1]. In addition, social interactions with the mother and experienced conspecifics may allow for a smooth transition to adapt to post-weaning feeding patterns [2]. In contrast, early weaning practices common in commercial pig husbandry occurring around 21–28 days of age depict an artificial scenario, in which piglets struggle to overcome the post-weaning adaptation syndrome with detrimental consequences on its subsequent performance [3,4,5]. Under such circumstances, feed consumption is well below the animal’s maximum capacity and is often unable to fulfill the nutrient requirements for optimal growth in piglets. Bruininx et al. 2001 [6] reported that approximately 45% of pigs do not start feed consumption until the first 15 h post-weaning. A nutritional strategy to promote an early feed consumption after weaning (e.g., by reducing the latency to first bite) is the use of highly palatable ingredients in the post-weaning diet, such as milk-derived products, or highly digestible cereal or animal origin protein sources [7,8]. Feed palatability may play a central role in increasing meal size and frequency particularly in early weaned pigs [9,10]. However, the manipulation of feed intake through palatability requires specific knowledge of several factors influencing taste [11].

The taste system in mammals, evolved to identify and incentive the consumption of nutritious foods, while avoiding potentially toxic compounds [11,12,13]. Pigs differentiate not less than five primary basic tastes: sweet, umami, sour, salty, and bitter [11]. Each one of these tastes is activated by different nutrients, such as simple carbohydrates that evoke sweetness, or L-amino acids and peptides that evoke umami [11,14]. The umami taste, first discovered by Ikeda in 1909 [15], is an innately hedonic taste in pigs related to dietary amino acids [11,14]. The main compound eliciting umami taste is L-glutamic acid, an acidic non-essential amino acid widely present in foods. Physiologically, glutamic acid has been described for having several roles in cell metabolism, including the participation in synthetic and oxidative pathways in tissues, and serving as energy substrate for the small intestine, among others [16,17]. The hedonic potential of glutamate (MSG) in pigs is enhanced by 5′-ribonucleotide monophosphates such as inosine or guanosine monophosphates [18]. The primary porcine umami receptor is the heterodimer pT1r1/pT1r3. However, additional receptors are also involved in umami taste sensing in pigs, such as the metabotropic glutamate receptors mGluR1 and mGluR4, which may respond exclusively to glutamic acid [11,14,19].

On the other hand, it is already known that maternal experiences during gestation and lactation have an impact on the progeny and its subsequent development [20]. During pregnancy, the human fetus becomes familiar with external sounds, as well as interacting with other sensory stimuli such as smell and taste [21,22]. In particular, the amniotic fluid in which newborns develop contains flavors derived from foods eaten by the mother [22]. Some of these flavors are also later transmitted to the milk, so that fetuses and lactating animals experience the flavors of their mother’s diet before their first exposure to these flavors in solid feeds [21,22,23,24]. Flavor transference and continuity has been described in several mammalian species beside humans, such as ewes [25], rabbits [26], rats [27], and pigs [28]. It has gained attention in an attempt to find strategies to improve dietary habits in humans, and in pigs related to the improvement of feed intake at weaning. Thus, there are a number of studies of flavor continuity in swine, mostly published during the last 10 years [28,29,30,31,32,33,34,35]. In general, they provide evidence for a reduction of stress around weaning, an increase in post-weaning performance and improvements in animal welfare. However, most of the work in pigs has been around feed volatiles, as opposed to taste active compounds. To our knowledge, there is no evidence that taste active compounds included into maternal diets may similarly exert an influence on the sensitivity of the same taste in young animals after weaning. In the present work, we hypothesized that the inclusion of MSG in maternal gestating and lactating diets would modify the feeding behavior of post-weaning pigs. The aim was therefore to assess changes in preference thresholds, sensory-motivated intake, total consumption, and consumption patterns of animals as dependent of a pre and postnatal inclusion of MSG into sows’ diets.

## 2. Materials and Methods

All procedures described in this study were conducted in a commercial pig farm located in the VI region of Chile (Libertador General Bernardo O’Higgins) in the central zone of the country (34°21′ S, 71°18′ W), between November 2017 and September 2018. Experimental procedures were approved by the Animal Use and Care Ethical Committee of the University of Chile (certificate N° 17018-VET-UCH).

### 2.1. Gestation Period

In total, 22 sows (Landrace × Large White) and 208 male and female piglets (PIC^®^337 × (Landrace × Large White)) born from these sows were selected to be used. On day 85 of gestation, sows were selected based on similar parity number (2.6 ± 0.8 (mean ± S.D.) parities), body condition (3.5 ± 0.6), and back fat thickness (9.6 ± 2.2 mm). Sows were then randomly divided into two experimental groups, consisting of a standard commercial feeding program (gestation and lactation diets) without (control) or with (MSG) dietary inclusion of 50 g/kg of MSG (Prinal S.A.; Santiago, Chile). The commercial feed was formulated to provide a complete and equilibrated nutrient content in order to fulfill the requirements of sows in each productive stage according to NRC [36] (Table 1). In the case of MSG-fed sows, MSG was directly and homogeneously included within the complete diet without manipulation of feed formulation. The diets were offered individually for each sow in mash form following a restriction pattern during gestation and an ad libitum regime during lactation period.

Body condition and back fat thickness of control and MSG-fed sows were also recorded on days 100 and 114 of gestation in order to assess possible modifications.

### 2.2. Lactation Period

At farrowing and during the lactation period (21 days), piglets born from each sow were identified by colored ear tags and remained with their respective mothers. Cross-fostering was available only between animals from the same experimental group. A commercial creep-feed was offered to all piglets starting from day 10 of life.

#### Milk Sampling

Six milk samples were collected from three different sows of each experimental group with different parity number (1 sample/sow), by using an adaptation of the method described by Peters et al. 2010 [37]. An intramuscular injection of 20 IU of oxytocin (Veterquímica S.A.; Santiago, Chile) was administered on day seven after farrowing. After 10 min of injection, approximately, a manual stimulation of mammary glands started. Milk was collected from all functional glands and was placed into 50 mL sterile polypropylene containers, with a minimum of 10 mL of milk per sample. They were then frozen and stored at -20 °C prior to chemical and amino acid analysis. Samples were analyzed using standard AOAC methods [38] for contents of moisture, crude protein, ether extract, and ash. Amino acids were determined after conventional hydrolysis according to White et al. 1986 [39]. Sample preparation and chromatographic conditions were also according to White et al. 1986 [39].

### 2.3. Weaning

Piglets were weaned on day 21 of life, when they were transferred to a nursery room with a total of 24 pens. Two-hundred and eight of the pigs born from control and MSG-fed sows were selected, 104 animals from each experimental group, and were allotted into eight nursery pens (26 pigs/pen, 4 pens/group). Pens were sized 2.8 m × 3.5 m (length × width) and were equipped with a feeder of 1.05 m × 30 cm with eight feeding spaces and three drinkers. During the first four days after weaning, pigs recovered from weaning stress and got accustomed to the room. After adaptation to the new environment, the pigs were trained to perform preference tests.

### 2.4. Training to Preference Tests

Pigs were trained in performing preference test procedures on days five and six after weaning, by using a variation of the methodology described by Roura et al. 2011 [40]. The 26 pigs from each pen were separated alternately within the same pen into 13 randomly determined pairs of animals by means of a removable dividing fence. Thus, each pair of pigs had an exclusive training area of 2.8 m × 1.0 m approximately. Each pair was simultaneously offered two drinkers, containing either 500 mL of tap water or 500 mL of a 200 mM sucrose solution. The drinkers were located at the center of the training area in an equidistant point from the midline at the left and the right, whose positions were determined at random. Training sessions were once a day for each pair of pigs and lasted 10 min on the first day and 5 min on the second day. The objective was that animals progressively realized that the two drinkers offered a choice of options that differed in hedonic value.

### 2.5. Preference Thresholds

After training, preference thresholds of pigs for MSG and sucrose solutions were determined in animals born from control and MSG-fed sows. Monosodium glutamate concentrations evaluated were 0.1 mM, 0.5 mM, 1 mM, 3 mM, 9 mM, and 27 mM. On the other hand, sucrose concentrations evaluated were 0.03 mM, 0.1 mM, 1 mM, 6 mM, 12 mM, and 18 mM. These concentrations were selected as they are close to the detection zone of each taste active compound, according to previous studies [14]. Tests consisted of short-duration preference tests (2 min) as previously described [41,42,43,44]. In these, each pair of pigs was offered two drinkers in the testing area, one containing plain water and the other containing the experimental MSG or sucrose solutions under evaluation. Five-hundred mL were offered in each drinker, and after 2 min of exposure they were removed, and the remaining content recorded. The consumption for each drinker was calculated by subtracting the remaining solution to the initial 500 mL. Preference values were calculated as the percentage of intake of the treatment solution (MSG or sucrose) relative to the total fluid intake from the two drinkers (treatment and water) and were compared to the neutral value of 50% (no preference). The preference threshold was then determined as the lowest concentration of MSG or sucrose to reach a preference value significantly greater than 50%.

### 2.6. Sensory-Motivated Intake

The sensory-motivated intake of a compound is defined as the amount of the test solution consumed above water and is calculated by a direct subtraction of the consumption from the test minus the control drinkers [45]. The lowest concentrations of MSG and sucrose to reach a sensory-motivated intake value significantly greater than 0 were used for comparison between pigs born from control and MSG-fed sows. The sensory-motivated intake may add fundamental information regarding the potential appetite enhancing of sapid compounds such as sucrose or MSG.

### 2.7. Total Consumption and Consumption Patterns

The total consumption and consumption patterns of MSG and sucrose solutions in pigs born from control and MSG-fed sows was later assessed in this experiment. To assess the total consumption, a single drinker containing 500 mL of solution was offered to each pair of animals for 2 min. Concentrations used in these tests were 1 mM, 3 mM, 9 mM, and 27 mM of MSG; and 1 mM, 6 mM, 12 mM, and 18 mM of sucrose. The consumption was calculated by subtracting the remaining solution to the initial content.

In order to quantify the consumption patterns, pigs were recorded by means of eight video cameras (1 camera/pen; SENKO S.A.; Santiago, Chile) placed in front of the pens to allow behavioral sampling during total consumption tests (2 min). Consumption time (total time in seconds drinking at the drinker) and approaches (number of times the drinker was approached with a consumption result) were assessed from the video recordings over the testing period. Consumption patterns were then calculated by dividing consumption time per number of approaches, analogous to the licks/bout measure used in rodents in lick cluster size analysis [46,47] that was recently proposed in pigs [48,49].

### 2.8. Statistical Analysis

Mean preference and sensory-motivated intake values for MSG and sucrose solutions were compared to the neutral values of 50% of preference and 0 intake, respectively, using a Student’s t-test through the MEANS procedure of SAS (9.4; SAS Inst. Inc.; Cary, NC, USA), considering experimental group (MSG inclusion in sows’ diets) as a main effect. Each pair of pigs was considered the experimental unit. The ANOVA was performed for total consumption and consumption patterns by using the GLM procedure of SAS, taking into account the experimental group as main factor. Milk data was also analyzed by parity number (2, 3, or 4 parities). For all of the analysis, average values were compared by least-squares means with the Tukey adjustment for multiple comparisons. The alpha level used for the determination of significance was 0.05. Statistical trends for 0.05 < *p* < 0.1 are also presented.

## 3. Results

### 3.1. Milk Composition

The chemical and amino acid composition of the milk samples collected from control and MSG-fed sows are shown in Table 2. No differences (*p* > 0.05) were determined in milk composition of control and MSG-fed sows according to their contents of moisture, crude protein, ether extract, N-free extract, ash, and energy. A tendency (*p* = 0.074) to a higher content in the N-free extract was observed in the milk of MSG-fed sows compared to sows fed standard diets. No effect (*p* > 0.05) of the parity number of sows was determined among these parameters. In relation to the amino acid profile, no differences (*p* > 0.05) were detected in the amino acid contents between the two groups. No differences (*p* > 0.05) in milk composition were observed relative to the number of parities.

### 3.2. Preference Thresholds

Table 3 shows the mean preference values obtained for MSG and sucrose solutions in post-weaning pigs from control or MSG-fed sows. In pigs born from control sows, the preference threshold for MSG was determined at 1 mM (*p* < 0.001). In contrast, the preference threshold for MSG was observed at 0.1 mM in pigs born from MSG-fed sows (*p* = 0.045). Regarding sucrose, the threshold was stablished at 12 mM in animals coming from control sows (*p* = 0.003). In contrast, the threshold for sucrose in pigs coming from MSG-fed sows during gestation and lactation was found to be 1 mM (*p* = 0.032).

### 3.3. Sensory-Motivated Intake

Sensory-motivated intake values generated by MSG and sucrose solutions in pigs born from control or MSG-fed sows are shown in Table 4. The MSG sensory-motivated consumption was significantly higher than water consumption in concentrations above 1 mM in pigs born from control sows (*p* < 0.015). In contrast, in pigs born from MSG-fed sows, the only concentration of MSG resulting in a significantly (*p* = 0.017) higher intake than water was 3 mM. Significantly higher sucrose consumption related to water was observed only at 12 mM in pigs born from control sows (*p* = 0.005). Conversely, in pigs born from MSG-fed sows, concentrations above 1 mM generated sucrose consumptions significantly higher than the negative control (*p* < 0.050).

### 3.4. Total Consumption

In single option total consumption tests, no significant differences (*p* > 0.058) were observed in the consumption values of MSG or sucrose solutions between pigs born from control or MSG-fed sows (Table 5). Pigs born from MSG-fed sows showed a tendency to a lower MSG intake than did animals born from control sows at 1 mM (*p* = 0.058) and 3 mM (*p* = 0.062).

### 3.5. Consumption Patterns

Consumption time, number of approaches, and consumption patterns of pigs born from control or MSG-fed sows during the 2-min exposure to MSG and sucrose solutions are shown in Table 6. Pigs coming from control sows exhibited a higher consumption time (*p* = 0.038) and number of approaches (*p* = 0.024) than did animals coming from MSG-fed sows at 1 mM MSG. However, no difference in the consumption pattern was observed between both groups of animals at this MSG concentration (*p* = 0.610). Similarly, no differences in consumption time (*p* > 0.125), approaches (*p* > 0.452) or consumption patterns (*p* > 0.143) were found at 3 mM, 9 mM, or 27 mM MSG among pigs born from control or MSG-fed sows. The same situation was observed with sucrose, since no differences were registered between pigs from the control or MSG-fed sows in consumption time (*p* > 0.212), approaches (*p* > 0.444), or consumption patterns (*p* > 0.275) at any concentration tested (1 mM, 6 mM, 12 mM, or 18 mM) over 2 min.

## 4. Discussion

The early weaning practice common in modern intensive pig production systems, represents one of the most critical phases because piglets are subjected to social, environmental, and nutritional stressors [4,5]. These include the abrupt separation from the sow at 21–28 days-of-age, transportation to a new facility, and the establishment of new social hierarchies [4]. In addition, pigs face a major nutritional stress as they must adapt from highly digestible and palatable liquid milk to a solid dry diet, less digestible and palatable [4]. As a result, pigs at weaning show reduced solid feed intake and growth performance. Under such scenario, the present work was conducted to test the hypothesis that the inclusion of MSG, an umami taste compound, in gestating and lactating diets for sows would modify the feeding behavior of nursery pigs. A better understanding of the factors driving voluntary feed intake and diet selection in pigs at this stage may contribute to the design of feeding strategies to promote an early post-weaning feed consumption.

It was observed that pigs born from MSG-fed sows showed a lower preference threshold for MSG (0.1 mM) in comparison to pigs born from control sows (1 mM), indicating that the sensitivity for umami increased 10 times in animals whose mothers were fed diets included with 50 g/kg of MSG during gestation and lactation. These results prove the concept that feeding behavior of post-weaning pigs can be influenced by pre and postnatal inclusion of a taste active compound into maternal diets. Previous studies have already demonstrated the high gustatory preferences of pigs for umami tastants [11,14,19,42], as well as the transference and continuity effect of different flavors included in the diets of sows to the progeny [28,29,30,31,32,33,34,35]. While most pig studies have focused on feed volatile compounds and/or feed flavors, emphasis here was on the effect of a sapid water-soluble compound such as MSG. The observed preference threshold for MSG in pigs born from control sows showed just 3 mM difference with a reference threshold previously reported by Roura et al. 2016 [14], who informed that nursery pigs without previous maternal experience with MSG showed a threshold for this compound at 3 mM. Thus, the results obtained under commercial conditions are consistent with previous knowledge developed under experimental conditions. We were also interested to assess the effect of MSG inclusion in gestating and lactating diets of sows on the sensitivity of pigs for other taste qualities other than umami. Sucrose is a disaccharide that elicits a hedonic taste (described as sweet in humans) in mammalian species including pigs [11,14,19,43]. Similar to the MSG response, pigs born from MSG-fed sows showed a lower preference threshold for sucrose (1 mM) compared to the control group (12 mM), reflecting a 12-fold increase in sweet sensitivity. Earlier work reported a sucrose preference threshold at 6 mM in animals without manipulation of maternal diets [14]. Thus, these results may indicate an umami-sweet taste interaction occurring in pigs regarding the pre and postnatal high-concentration umami inclusion and the post-weaning sweet sensitivity threshold [50,51]. Umami taste in pigs has been related to the sensing of dietary amino acids while sweet taste to simple carbohydrates. Thus, the results enhancing the sensitivity for both amino acids and energy seem to respond to a nutrient balance principle aimed at maintaining a narrow range of protein-to-energy ratios in order to gain optimal growth and gut development [52].

The sensory-motivated consumption of MSG and sucrose solutions was also assessed in this study. Given that MSG and sucrose were tested in water solution, and pigs had ad libitum access to water, their desire to drink during the choice scenario might have been mainly a function of the appetite linked to that solution. In pigs born from MSG-fed sows, it was observed that MSG solution was unable to promote a significant consumption at concentrations different from 3 mM, while MSG-driven consumption was significantly higher from that of water at concentrations above 1 mM in animals born from control sows. On the contrary, an opposite behavior, and closely related to preference thresholds outcomes, was registered regarding sucrose-motivated intake among both groups of pigs. Pigs born from MSG-fed sows showed sucrose consumptions significantly higher than water at concentrations higher than 1 mM. In contrast, pigs born from control sows did not display significant sucrose intakes until 12 mM concentration. This indicates that MSG inclusion during gestation and lactation not only increased the sensitivity of post weaning pigs for sweet solutions, but also it increased their sensory-motivated intake for sweet taste compounds during the nursery period.

In order to evaluate the effect of MSG supplementation on maternal fluids that could explain some of the behavioral changes previously described on the progeny, the milk chemical and amino acid profile of control and MSG-fed sows was assessed. However, it was observed that the inclusion of 50 g/kg of MSG did not modify milk composition of treated sows. Amino acid profiles were consistent with previous reports from Wu and Knabe 1994 [53] and Hou and Wu 2018 [54], indicating that glutamic acid is one of the most abundant free amino acids in sows’ milk to support growth of piglets. In particular, the lack of response to dietary glutamate was also consistent with previous reports showing that most of the dietary glutamic acid was catabolized in the small intestine of pigs and used as a major energy source for the gut [16,17,55]. In young animals approximately 97% of dietary glutamate is utilized by the small intestine, and only 3% enters the portal vein [16,56]. As suggested by Brosnan et al. 2014 [57], the gastrointestinal tract in animals and humans functions as a barrier to prevent the distribution of ingested MSG to the rest of the body. When free or protein-derived glutamate is absorbed from the intestinal lumen, it is oxidized by enterocytes to generate ATP, converted to other non-essential amino acids (e.g., alanine, aspartate) and other small molecules (e.g., glutathione), and used to synthesize intestinal proteins [57]. Therefore, even when MSG is ingested in large amounts such as in this study, it may have resulted in no or only a small transient increase in circulating levels of glutamate [16,55]. In addition, it has also been informed that the ingestion of MSG in maternal diets do not expose fetuses or newborn animals to increases in glutamate concentrations in biological fluids [57]. During gestation, the placenta is a natural barrier to the penetration of glutamate from the maternal into the fetal circulation, as it normally extracts glutamate from both maternal and fetal circulations to be used as energy substrate [57,58]. In relation to milk, studies in humans have shown that breast milk-free glutamate concentrations did not increase after the ingestion of a single dose of 150 mg/kg of MSG [57,59], which is similar to the results presented here with a much higher dose of supplemental MSG. All this evidence suggests that feeding behavior modifications observed in nursery pigs in this study do not seem to be related to a direct animals’ exposure to MSG during gestation. However, during lactation, piglets might be exposed to the sow’s diet and learn from the MSG supplement. The specific metabolic pathways from which MSG-derived metabolites influence feeding behavior patterns on the progeny require further research.

Even though MSG inclusion in prenatal and postnatal diets modified the preference thresholds and sensory-motivated intake of post-weaning animals, no effects on total consumption and consumption patterns of MSG or sucrose were observed after the short-term single-exposure to these solutions. One possible explanation could be attributed to that these measures were registered after the preference tests, consequently, later in pigs’ post-weaning life. This may have generated an attenuation in the influence of MSG-derived metabolites through maternal fluids. Another explanation may rely on the low MSG and sucrose concentrations used in these tests that did not allow us to observe significant differences between the two experimental groups. Previous studies in nursery pigs associated consumption patterns to palatability, or hedonic perception related to MSG that was directly linked to the concentration at lower suprathreshold levels while the consumption reach a plateau at higher concentrations [48,49]. However, these effects were determined with solutions that ranged between 0.1 mM to 300 mM. In contrast, the highest concentration tested in this study was 27 mM. The impact of prenatal and early postnatal experiences with specific taste qualities such as umami on the acceptance of the same taste later in life was systematically reviewed in children [60]. However, to the best of our knowledge, this is the first study to report the programing of the acceptability of MSG and sucrose.

## 5. Conclusions

It is concluded that feeding behavior of nursery pigs can be influenced by pre and postnatal inclusion of a taste active compound into maternal diets. This is reflected by lower preference thresholds for MSG and sucrose in pigs born from MSG-fed sows in comparison to pigs born from control sows. In addition, pigs whose mothers were fed diets included with 50 g/kg of MSG during gestation and lactation showed an increased sensory-motivated intake for sucrose compared to animals from the control group. In contrast, MSG supplementation decreased MSG-motivated intake and did not influence the total consumption and consumption patterns of pigs for these solutions. Monosodium glutamate inclusion in maternal diets modifies feeding behavior in pigs by increasing specific appetites for basic nutrients not present in the maternal diets such as increasing the liking for sucrose. Further research is required to elucidate the specific metabolic pathways from which MSG-derived metabolites influence feeding behavior patterns on the progeny.

## Figures and Tables

**Table 1:** Composition and chemical analysis of the gestating and lactating diets of sows used in the experiment.

Item	Gestation			Lactation	
	Control	MSG		Control	MSG
*Ingredients (g/kg)*					
Corn	572.0	572.0		594.7	594.7
Wheat meal	250.0	250.0		80.0	80.0
Soybean oil	-	-		40.0	40.0
Soybean meal	134.0	134.0		242.0	242.0
L-Lysine HCl	2.0	2.0		6.7	6.7
DL-Methionine	-	-		2.7	2.7
L-Threonine	-	-		2.8	2.8
L-Tryptophan	-	-		0.5	0.5
Mycotoxins inactivator ^1^	1.0	1.0		1.0	1.0
Mycotoxins absorbent ^2^	1.0	1.0		2.0	2.0
Sweetener ^3^	0.1	0.1		-	-
Mineral-vitamin-phytase mix ^4^	4.0	4.0		4.0	4.0
Calcium carbonate	12.0	12.0		10.0	10.0
Sodium bicarbonate	7.0	7.0		-	-
Dicalcium phosphate	7.0	7.0		7.0	7.0
Salt	5.0	5.0		5.0	5.0
Copper sulphate	-	-		0.5	0.5
Ammonium chloride	3.0	3.0		-	-
Vegetable choline chloride	2.0	2.0		1.0	1.0
MSG ^5^	-	50.0		-	50.0
*Chemical analysis (%)*					
Dry matter	87.6	89.3		89.3	89.7
Crude protein	15.4	19.7		18.7	19.9
Crude fiber	3.8	4.8		2.6	3.3
Ether extract	2.5	4.7		5.3	4.1
N-free extract	60.0	52.4		56.3	56.1
Ash	5.9	7.7		6.4	6.3

^1^ Micofix plus (Biomin; Getzersdorf, Austria); ^2^ Fintox mold plus (Lípidos Toledo S.A.; Madrid, Spain); ^3^ Sucram C-150 (Pancosma S.A.; Geneva, Switzerland); ^4^ Quantum blue (AB Vista Feed Ingredients; Marlborough, UK); ^5^ Monosodium glutamate (Prinal S.A.; Santiago, Chile).

**Table 2:** Effect of monosodium glutamate (MSG) inclusion into maternal gestating and lactating diets on chemical and amino acid composition of sows’ milk ^1^.

Item	Group			*p*-Value	
	Control	MSG	SEM	Group	Parity
*Chemical analysis (%)*					
Moisture	80.8	81.4	0.394	0.394	0.668
Crude protein	5.4	5.2	0.227	0.597	0.912
Ether extract	8.2	7.5	0.340	0.252	0.591
N-free extract	4.8	5.2	0.082	0.074	0.750
Ash	0.8	0.7	0.047	0.667	1.000
Energy (Kcal/100 g)	115.0	109.0	2.858	0.276	0.570
*Amino acid composition (mg/100 mL)*					
Aspartic acid	526.0	468.1	15.499	0.119	0.250
Glutamic acid	1272.5	1181.2	27.923	0.147	0.319
Serine	262.7	261.8	11.394	0.961	0.572
Glycine	164.4	148.2	6.149	0.202	0.801
Histidine	129.2	119.8	6.753	0.426	0.512
Arginine	206.9	201.0	7.585	0.638	0.228
Threonine	197.3	200.7	8.983	0.816	0.919
Alanine	211.0	230.2	15.967	0.485	0.637
Proline	485.7	452.8	19.447	0.355	0.984
Tyrosine	69.2	78.3	9.289	0.560	0.533
Valine	282.0	268.1	11.829	0.495	0.901
Methionine	53.1	63.7	2.654	0.106	0.173
Cysteine	19.9	20.0	1.638	0.980	0.231
Isoleucine	246.6	229.8	10.681	0.383	0.773
Leucine	449.0	435.0	20.482	0.677	0.974
Phenylalanine	207.4	202.0	13.158	0.799	0.947
Lysine	388.3	383.6	31.206	0.924	0.800

^1^ From ANOVA analysis, including the effects of experimental group (control (standard gestating and lactating diets) or MSG (gestating and lactating diets included with 50 g/kg of MSG)) and parity number (2, 3, or 4 parities). Treatment n = 3. Mean values are least-squares means with a significance level of *p* < 0.05. Six milk samples were collected on day seven after farrowing from three different sows of each group, following an intramuscular injection of 20 IU of oxytocin. Samples were analyzed for chemical and amino acid composition using standard AOAC methods [38] and White et al. 1986 [39].

**Table 3:** Preference values for different concentrations of MSG and sucrose solutions in post-weaning pigs born from control and MSG-fed sows ^1^.

Item	Control			MSG		
	Preference (%)	SEM	*p*-Value	Preference (%)	SEM	*p*-Value
*MSG concentration (mM)*						
0.1	51.5	7.31	0.845	57.8	3.47	0.045
0.5	57.6	5.98	0.229	62.5	6.46	0.079
1	68.5	3.17	<0.001	71.2	4.33	<0.001
3	63.3	4.75	0.016	66.9	5.72	0.012
9	68.4	4.88	0.003	59.2	7.66	0.253
27	68.6	4.26	<0.001	59.9	4.66	0.057
*Sucrose concentration (mM)*						
0.03	-	-	-	47.1	5.16	0.581
0.1	-	-	-	62.2	7.51	0.129
1	58.7	4.84	0.096	66.5	6.78	0.032
6	62.7	6.68	0.081	66.7	6.21	0.019
12	72.5	6.02	0.003	61.9	7.34	0.131
18	65.2	6.95	0.049	66.7	4.42	0.003

^1^ From Student’s t-test, including the effect of experimental group (control (pigs born from sows fed standard gestating and lactating diets) or MSG (pigs born from sows fed gestating and lactating diets included with 50 g/kg of MSG)). Treatment n = 13. Tests consisted of short-duration preference tests (2 min). The thresholds were identified as the lowest concentrations of MSG or sucrose resulting in preferences values significantly (*p* < 0.05) higher than 50%.

**Table 4:** Sensory-motivated intake of different concentrations of MSG and sucrose solutions in post-weaning pigs born from control and MSG-fed sows ^1^.

Item	Control			MSG		
	Sensory-Motivated Intake (g)	SEM	*p*-Value	Sensory-Motivated Intake (g)	SEM	*p*-Value
*MSG concentration (mM)*						
0.1	−9.3	29.69	0.760	35.3	17.73	0.070
0.5	70.6	36.38	0.076	57.3	34.05	0.121
1	47.9	16.97	0.015	23.2	32.17	0.485
3	85.6	28.97	0.012	80.6	28.94	0.017
9	122.9	38.36	0.008	63.7	62.24	0.327
27	127.4	32.15	0.002	58.4	37.93	0.150
*Sucrose concentration (mM)*						
0.03	-	-	-	-8.9	42.30	0.463
0.1	-	-	-	21.3	21.38	0.339
1	27.6	14.69	0.085	51.6	23.70	0.050
6	53.2	34.89	0.153	61.4	23.80	0.024
12	109.7	32.01	0.005	77.5	32.91	0.038
18	89.4	43.85	0.064	126.7	33.64	0.003

^1^ From Student’s *t*-test, including the effect of experimental group (control (pigs born from sows fed standard gestating and lactating diets) or MSG (pigs born from sows fed gestating and lactating diets included with 50 g/kg of MSG)). Treatment n = 13. Tests consisted of short-duration preference tests (2 min). Sensory-motivated intake was measured as the amount of MSG or sucrose consumed above water and was compared to the negative control (no intake).

**Table 5:** Pigs total consumption of different concentrations of MSG and sucrose solutions in animals born from control and MSG-fed sows ^1^.

Item	Intake (g)			
	Control	MSG	SEM	*p*-Value
*MSG concentration (mM)*				
1	216.5	140.7	26.91	0.058
3	245.7	167.1	28.37	0.062
9	294.0	248.7	30.03	0.297
27	257.6	273.2	28.21	0.699
*Sucrose concentration (mM)*				
1	173.1	134.9	23.11	0.253
6	172.9	191.8	26.63	0.621
12	197.0	185.3	27.3	0.755
18	237.3	226.4	28.42	0.790

^1^ From ANOVA analysis, including the effect of experimental group (control (pigs born from sows fed standard gestating and lactating diets) or MSG (pigs born from sows fed gestating and lactating diets included with 50 g/kg of MSG)). Treatment n = 13. Mean values are least-squares means with a significance level of *p* < 0.05. Tests consisted in offering a single drinker for 2 min.

**Table 6:** Consumption time, number of approaches, and consumption patterns of different concentrations of MSG and sucrose solutions in post-weaning pigs born from control or MSG-fed sows ^1^.

Item	Consumption Time (CT, s)				Number of Approaches (A)				Consumption Pattern (CT/A)			
	Group				Group				Group			
	Control	MSG	SEM	*p*-Value	Control	MSG	SEM	*p*-Value	Control	MSG	SEM	*p*-Value
*MSG concentration (mM)*												
1	35.5	24.8	3.47	0.038	9.5	7.5	0.60	0.024	3.7	3.5	0.31	0.610
3	35.9	27.7	3.66	0.125	9.7	8.8	0.82	0.452	3.7	3.2	0.22	0.143
9	42.3	39.7	4.48	0.683	9.7	9.5	0.66	0.840	4.4	4.3	0.49	0.865
27	43.0	40.6	4.34	0.706	9.2	9.7	0.71	0.573	4.9	4.1	0.38	0.143
*Sucrose concentration (mM)*												
1	30.4	24.7	3.14	0.212	8.5	7.9	0.68	0.582	3.6	3.1	0.29	0.277
6	26.5	29.8	4.08	0.571	7.3	6.8	0.81	0.668	3.7	4.5	0.51	0.275
12	29.9	31.4	4.12	0.804	8.5	8.5	0.71	0.940	3.5	3.8	0.47	0.708
18	35.5	35.2	5.00	0.974	8.4	9.2	0.70	0.444	4.0	3.8	0.37	0.825

^1^ From ANOVA analysis, including the effect of experimental group (control (pigs born from sows fed standard gestating and lactating diets) or MSG (pigs born from sows fed gestating and lactating diets included with 50 g/kg of MSG)). Treatment n = 13. Mean values are least-squares means with a significance level of *p* < 0.05. Pigs were recorded by 8 video cameras placed in front of the pens during total consumption tests (2 min). Consumption time (total time in seconds drinking at the drinker) and approaches (number of times the drinker was approached with a consumption result) were registered. Consumption patterns were calculated by dividing consumption time per number of approaches [48,49].
